# Valence-space associations in touchscreen interactions: Valence match between emotional pictures and their vertical touch location leads to pictures' positive evaluation

**DOI:** 10.1371/journal.pone.0199972

**Published:** 2018-07-18

**Authors:** Sergio Cervera-Torres, Susana Ruiz Fernández, Martin Lachmair, Peter Gerjets

**Affiliations:** 1 Leibniz-Institut für Wissensmedien, Tübingen, Germany; 2 LEAD Graduate School and Research Network, University of Tübingen, Tübingen, Germany; 3 FOM-Hochschule für Oekonomie und Management, Stuttgart, Germany; Boston Children's Hospital / Harvard Medical School, UNITED STATES

## Abstract

Embodied cognition research suggests that bodily experiences might ground mental representations of emotional valence in the vertical dimension of space (i.e., positive is up and negative is down). Accordingly, recent studies show that upward and downward arm movements may also influence the evaluation of valence-laden stimuli, suggesting that upward (downwards) movements lead to more positive (negative) evaluations. Interestingly, these studies typically did not investigate paradigms that require a direct hand interaction with the stimuli. With the advent of touchscreen devices and their use for experimental environments, however, a direct and more natural hand interaction with the stimuli has come to the fore. In this regard, the goal of the present study is to examine how direct hand interaction with valence-laden stimuli on a touchscreen monitor affects their perceived valence. To do so, participants evaluated emotional pictures after touching and moving them either upwards or downwards across a vertically mounted touchscreen. In contrast to previous findings, the results suggest that positive pictures were evaluated as more positive after downward movements while negative pictures were evaluated as less negative following upward movements. This finding may indicate that a matching between the pictures’ valence and the valence associated with their vertical touch location leads to more positive evaluations. Thus, the present study extends earlier results by an important point: Touching the emotional pictures during movement may influence their valence processing.

## Introduction

Colloquial expressions like “jumping up for joy” or “feeling down” are commonly used to express positive or negative emotional states. Such metaphorical expressions might serve to facilitate the mental representation of more abstract concepts like happiness or sadness by grounding them into bodily experiences in the vertical space [1,2]. Bodily experiences such as upright body postures, for instance, are usually associated with positive emotions compared to slumped postures, which are associated with negative emotions [3,4]. Accordingly, positive concepts (e.g., “joy”) are associated with the upper space and negative concepts (e.g., “sadness”) are associated with the lower space [5]. There is converging evidence for this metaphorical mapping. For example, it has been shown that participants evaluated the valence of affective words faster when positive words (e.g., “brave”) were presented in an upper compared to a lower location on a monitor screen. The opposite held for negative words (e.g., “poison”) with faster evaluations in a lower compared to an upper location on the screen [6,7]. This suggests that mental representations of positive and negative valence are strongly associated with representations of vertical space considering positive-up and negative-down as congruent valence-space mappings resulting in shorter response latencies, but positive-down and negative-up as incongruent valence-space mappings that result in longer response latencies.

In addition to these findings, other studies indicate that the processing of valence words can also affect subsequent motor responses performed across the vertical space. Typically, responses upward are faster after processing positive stimuli whereas responses downward are faster after processing negative stimuli. For instance, Brookshire, Ivry, and Casasanto [8] designed a study where a positive or negative word appeared on the center of a computer screen after participants pressed a centered key on a vertically mounted keyboard. Participants were instructed to release this key and to press subsequently a key located either at the top or the bottom of the keyboard by performing an upward or downward directed arm movement. The results indicated that participants responded faster upwards when the presented word was positive and faster downwards when the presented word was negative. These findings suggest that the processing of affective stimuli facilitates the performance of arm movement responses with a congruent valence-space mapping, but on the contrary, hinders arm movement responses with an incongruent valence-space mapping.

Against this background, one can state that research in this field has examined two important questions. The first addressed the processing of affective concepts located in vertical space [6,7], and the second addressed motor responses in vertical space subsequent to the processing of affective stimuli [8,9,10]. However, these studies typically used traditional experimental setups with computers and monitors, vertically mounted keyboards or connected external keys as response devices, but they do not involve direct interaction with the presented stimuli. Interestingly, with the advent of human-computer interfaces that are based on touch interactions, the findings of these studies may appear in a new light. When using touchscreen devices, emotional stimuli displayed on the screen are now moved by means of touch and rather natural moving hand or arm gestures [11, 12]. In contrast to traditional setups, this means that the stimuli stick to the acting hand or finger during the whole (inter-)action. Indeed, this form of *direct interaction* is nowadays an everyday experience and became standard to a large part of human beings when using smartphones, touchpads, etc. Thus, from a psychological perspective that considers body states and actions as relevant for cognitive processes, it seems relevant to investigate this form of interaction with valence-laden stimuli in more detail. However, so far, studies on this question are rare.

One study that points in this direction is the study by Sasaki, Yamada, and Miura [13]. In their study, participants were presented with emotional pictures on a touchscreen device. The pictures were presented for 500ms at the center of the screen. Then a dot appeared together with an action cue indicating whether the dot had to be moved towards an upper or lower location of the screen. After moving the dot towards the indicated location by performing an upward or downward arm movement, participants had to evaluate the picture. The crucial finding was that the evaluation of the pictures was influenced by the movement direction only if the movement was performed immediately after the presentation of the picture. Concretely, the valence of the pictures was more positive subsequent to upwards directed arm movements but more negative subsequent to downwards directed arm movements.

The authors explained this effect with the space-valence metaphor mapping stating that an upward arm movement is metaphorically associated with positive valence and a downward arm movement with negative valence. They concluded that when a movement metaphorically associated with an emotion is performed immediately after the presentation of an emotional picture, the emotion of the movement retrospectively modulates the emotional valence of the pictures. However, although this study uses a touchscreen device, it has not examined moving gestures where the finger sticks to the affective picture, which is in fact, standard in the use of touch devices. This could be a critical issue, given that the distance between the picture and the interacting hand can influence the processing of the stimulus (e.g., [14]). The study by Brucker, Ehrmann, and Gerjets [15], for example, showed that properties of stimuli that were presented near the hand were better remembered than properties of stimuli presented far from the hand. Thus, it is unclear if performing a vertical movement with a picture that sticks to the hand causes similar influences on cognitive processes than performing the movement without the picture, as is the case in rather artificial or traditional experimental setups.

Therefore, building upon these findings, the present study is concerned with the question of how the standard use of a touchscreen device through bodily interaction can affect the processing of positive and negative valence-laden pictures when touched and moved along a vertical spatial axis on the device. To investigate this issue, participants were instructed to move emotional pictures on a vertically mounted touchscreen monitor. The movements were performed either from a starting location on the upper edge towards an end location at the lower edge (i.e., downward directed movement), or from a starting location on the lower edge of the screen towards a location at the upper edge (i.e., upward directed movement). Afterwards, they were instructed to evaluate the valence of the just moved pictures.

In principle, two different expectations can be formulated according to this setting: First, if touching the pictures during performing the moving gesture is irrelevant for judging their valence, similar results than in the study by Sasaki et al. [13] can be expected. Accordingly, the moving direction of the picture should influence its evaluation regardless of its valence, which means that both, positive and negative pictures should be evaluated more positively when moved upwards and more negatively when moved downwards. But second, if touching the pictures during performing the moving gesture is relevant for judging their valence, a different outcome can be expected. In this case, the valence of the picture that sticks to the hand could get into the focus of attention together with the valence category of the surrounding space. This could promote a link between the valence of the picture (i.e., positive and negative) and the valence of its touch location (i.e., upper space-positive and lower space-negative). In this line of reasoning the promoted link can represent a match between the valences of the picture and the touch location (i.e., a positive picture in a positive upper location and a negative picture in a negative lower location) or a mismatch (i.e., a positive picture in a negative lower location and a negative picture in a positive upper location). Critically, former findings suggest that a match between the valences of affective stimuli and their spatial locations can be perceived as congruent and thus evaluated more positively compared to a mismatch. To put things more concrete, Schnall and Clore [16] reported that positive words (e.g., kindness) presented at the upper side of a sheet of paper and negative words (e.g., garbage) presented at the lower side, were both rated as more positive. In contrast, positive words presented in the lower side and negative words presented in the upper side) were rated as more negative. In other words, the matching or congruence between the valence of the stimuli and the valence of its location lead to more positive evaluations compared to the mismatching or incongruence between the valence of the stimuli and the valence of its location (cf. [17]).

According to these findings, the results of the present study should show that positive and negative pictures would be evaluated more positively if they were moved from a congruent to an incongruent location with regard to the associated valence. This means that moving positive pictures on a touchscreen from top-to-bottom or negative pictures from bottom-to-top would result in a relatively positive evaluation. In contrast, moving positive pictures from bottom-to-top or negative pictures from top-to-bottom would result in a relatively negative evaluation.

## Materials and methods

### Participants

The experimental testing was in agreement with the guidelines for good scientific practice at the University of Tübingen (Germany) and in accordance with the 1964 Helsinki declaration and its later amendments. In addition, the ethics committee of the Leibniz-Institut für Wissensmedien of Tübingen has approved the procedure used in the study. Participants' anonymity was always preserved. At no point the recorded data could be associated with a participant's name. The manuscript does not contain clinical studies or individuals´ baseline demographic data other than age and gender. Written informed consent was obtained from all participants prior to their participation in the study. Participants were recruited between October the second and December the eighteenth of 2015 by means of the Online Recruitment System for Economic Experiments (ORSEE [18]). Participants received financial reimbursement of 8 Euros for participation. Right-hand dominance was set as the main experimental inclusion criteria.

Eighty-six participants signed up in the ORSEE for participation in the study 25% did not participate in the experiment due to their own decision; further 5% could not participate due to left-handedness. Accordingly, a total of 60 right-handed participants with ages ranged between 19 and 33 years old (*M*_age_ = 23.8, SD = 3.2; 75% women) took part in the experiment. The Edinburgh Inventory by Oldfield [19] modified by Salmaso and Longoni [20] was used to provide information about participants’ handedness. All participants had normal or corrected-to-normal vision.

### Apparatus and stimuli

A vertically mounted touchscreen monitor (TM; Dell™-Monitor S2340T) connected to a computer (Lenovo ThinkPad T410, Intel Core i7 620M, 2.67 Ghz) was used to display the stimuli. The TM was of 23 inch (20.99" (V) x 12.28" (H)) Activ-Matrix-TFT-LCD and featured a resolution of 1600 x 900 pixels.

Forty pictures, twenty positive and twenty negative from the International Affective Picture System (IAPS; [21]), were used as stimuli (Table 1). An ANOVA on the pictures’ valence means confirmed differences between the valence categories, *F*(1, 38) = 595, *p* < 0.001. Pictures’ arousal means, on the contrary, did not show significant differences, *F*(1,38) = 1.46, *p* = 0.15. Pictures were presented on the TM with a resolution of 397 x 340 pixels (10.5 cm x 9 cm), at a vertical distance of 42 cm to a white square (6.2 cm x 6.2 cm).

**Table 1:** The table shows the affective pictures of the international affective picture system (IAPS) used in the present study. Code, description, valence, and arousal are provided for each positive and negative picture. The pictures can be retrieved upon request at the following website http://csea.phhp.ufl.edu/media/iapsmessage.html.

IAPS Positive Category				IAPS Negative Category			
Code	Description	Valence	Arousal	Code	Description	Valence	Arousal
1340	Women	7.13	4.75	1111	Snakes	3.25	5.20
1811	Monkies	7.62	5.12	1270	Roach	3.68	4.77
1920	Porpoise	7.90	4.27	1274	Roaches	3.17	5.39
1999	Mickey	7.43	4.77	2120	Angry face	3.34	5.18
2154	Family	8.03	4.48	2141	Grieving	2.44	5.00
2209	Bride	7.64	5.59	2205	Hospital	1.95	4.53
2311	Mother	7.54	4.42	2375.1	Woman	2.20	4.88
2340	Family	8.03	4.90	2692	Bomb	3.36	5.35
2346	Kids	7.05	5.28	2710	Drug addict	2.52	5.46
2352	Kiss	6.94	4.99	2800	Sad child	1.78	5.49
2362	Girl & dog	6.74	4.60	3350	Infant	1.88	5.72
2373	Band	6.97	4.50	6242	Gang	2.69	5.43
2391	Boy	7.11	4.63	9000	Cemetery	2.55	4.06
2398	Boat	7.48	4.74	9090	Exhaust	3.69	4.80
2550	Couple	7.77	4.68	9280	Smoke	2.80	4.26
2900.2	Smiling girl	6.62	4.52	9342	Pollution	2.85	4.49
4250	Attractive female	6.79	5.16	9417	Ticket	3.16	4.83
4520	Erotic male	6.16	4.80	9440	Skulls	3.67	4.55
5628	Mountains	6.51	5.46	9560	Duck in oil	2.12	5.50
8500	Gold	6.96	5.60	9911	Car accident	2.30	5.76
Mean (SD)		7.22 (.53)	4.86 (.39)			2.77 (.53)	5.03 (.49)

### Procedure

**Fig 1:**
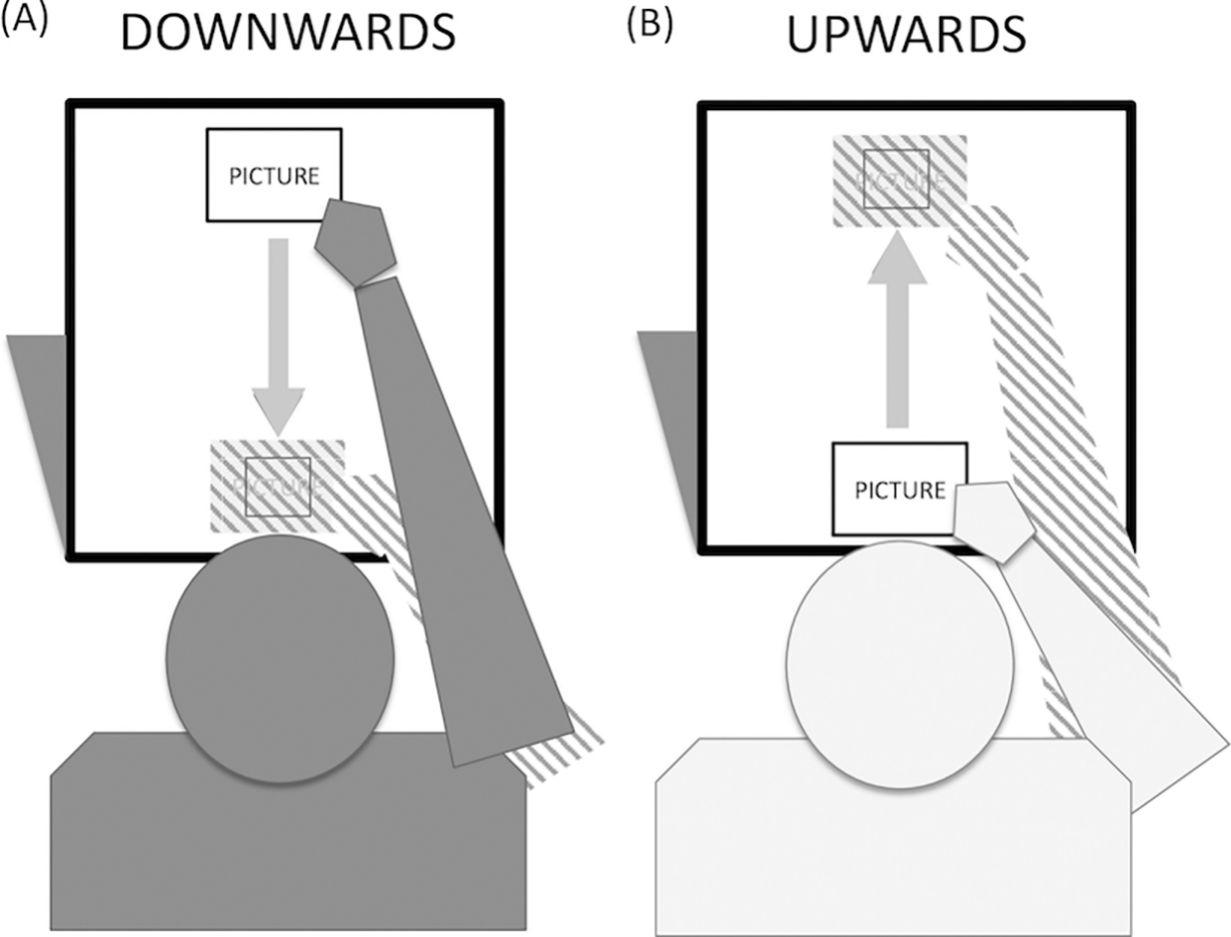
The figure shows two experimental groups. (A) Participants of this group (n = 30) touched and subsequently moved the pictures from the top to the bottom of the TM (i.e., downward movement). (B) Participants of this group (n = 30) touched and subsequently moved the pictures from the bottom to the top of the TM (i.e., upward movement). Dashed areas represent the final arm and picture position.

In order to control for the differences within and between the experimental groups, participants evaluated the valence of the pictures 48 hours prior to the experiment. Then participants were randomly assigned to one of two experimental groups. In one group, participants had to touch and move the randomly presented pictures with their right hand from the top of the TM towards an empty square located at the bottom of the TM (i.e., downward movement condition). In the other group, participants had to touch and move the pictures with their right hand from the bottom of the TM towards an empty square located at the top of the TM (i.e., upward movement condition; Fig 1). Afterwards, the just moved picture was again presented in the middle of the TM together with a 9-point Likert scale below for valence evaluation (i.e., 1 –negative valence, 9 –positive valence). After this evaluation, the next trial began. The presentation order of the pictures was randomized. A description in more detail of this procedure can be found here http://dx.doi.org/10.17504/protocols.io.qagdsbw.

### Data analyses

All analyses were performed by means of linear mixed-effects models (LMM) via maximum likelihood with the software package SPSS 21.0 (IBM). This method has been found appropriated to simultaneously control the error variance with regard to participants, stimuli and/or participant x stimuli interactions [22,23]. Valence evaluations were analysed with a 2 (Movement type: upward vs. downward) x 2 (Valence category: positive vs. negative) fixed-effects structure. Movement was manipulated between participants whereas valence category was manipulated within participants. Valence evaluations of the baseline were used as a control covariate. Participants and pictures were included as random factors with random intercepts in order to account for between-participants and between-pictures differences on the valence evaluations. To calculate *p*-values estimates for the fixed-effects, a Type III Satterthwaite approximation was used [24,25].

## Results

**Fig 2:**
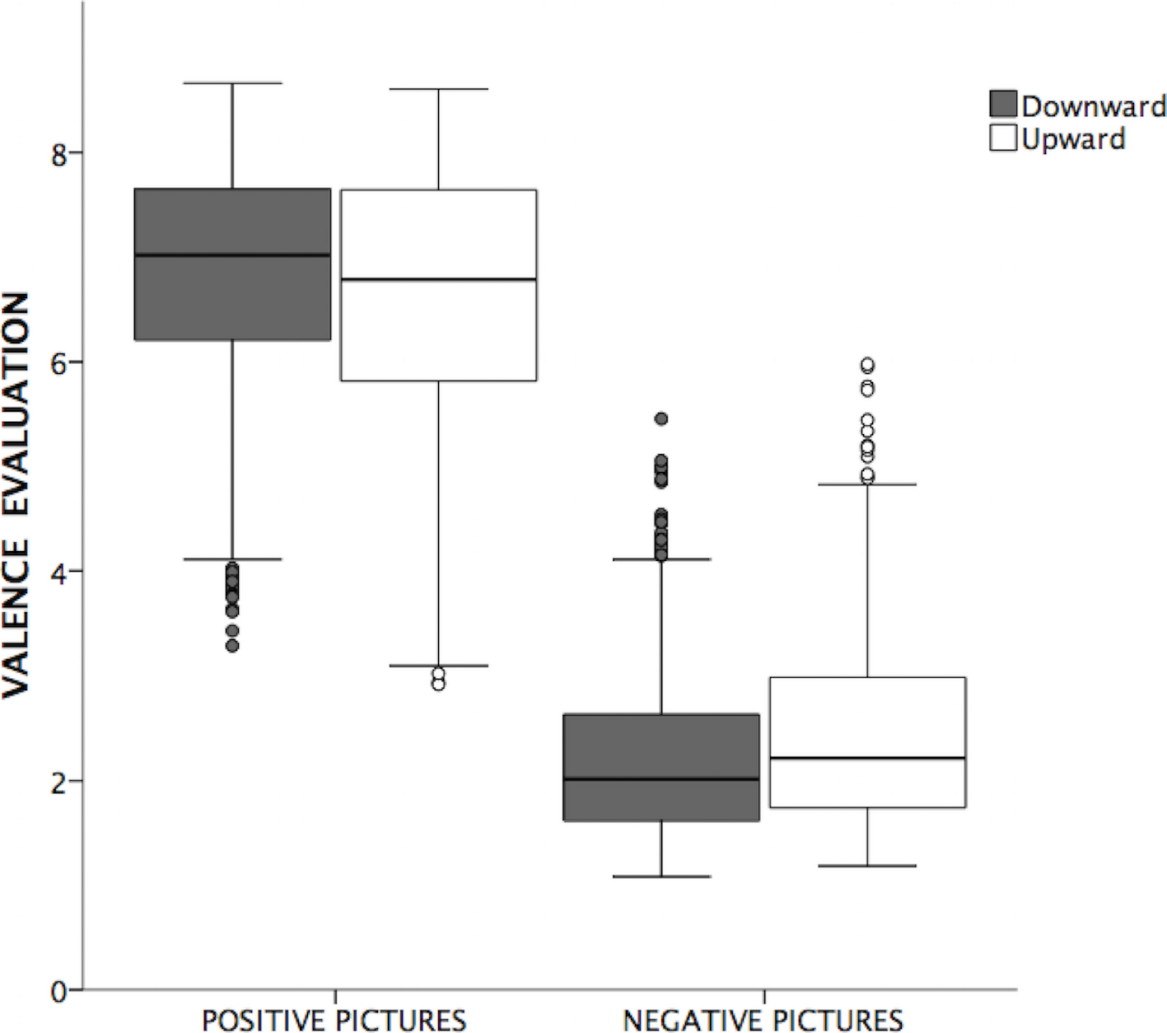
Valence evaluations of positive and negative pictures after being moved downward or upward.

The modelling of the resulting data reached better goodness of fit (*AIC* = 7718.15) compared to a model with only random factors, (*AIC* = 8790.61), *χ*^*2*^ = 13.28, *p* < 0.01. Specifically the analysis showed a significant main effect of valence category, *F*(1,89) = 314, *p* < 0.001, indicating that positive pictures were evaluated more positively than negative pictures. There was no significant main effect of movement, *F*(1,59) = .015, *p* = 0.903, indicating that movement type had not an overall influence on the evaluation of the pictures. Interestingly, the interaction between vertical movement and valence category was highly significant, *F*(1,2281) = 15.7, *p* = 0.001 (Fig 2). Post-hoc analyses of this interaction using Bonferroni correction revealed that:

a) Positive pictures moved downwards were evaluated more positively than positive pictures moved upwards, *t*(108) = 2.36, *p* = 0.020, d = .54.
b) Negative pictures moved upwards were evaluated less negatively than negative pictures moved downwards, *t*(129) = 2.02, *p* = 0.045, d = .36.

This result-pattern suggested that positive and negative pictures resulted in more positive valence evaluations when they were touched and subsequently moved from their congruent spatial locations (i.e., positive-up; negative-down). However, post-hoc diagnostics on the analysis’s residuals revealed the presence of extreme observations, which potential influence on the robustness of the results had to be inspected (e.g., positive pictures rated very negatively or negative pictures rated very positively). To examine this question, we turned to a multiverse analyses approach by which the results are rather interpreted in terms of observed trends on the data [26, 27]. Specifically, to deal with the extreme observations, we carried out LMM analyses according to the following criteria: a) a logarithmic Box-Cox transformation of the valence ratings, b) exclusion of valence ratings greater or less than 3, 2.5, and 2 SDs from each participant average in the positive and negative pictures’ categories, and from each picture average in each experimental group, c) exclusion of valence ratings greater than 2.5 median absolute deviations (MAD; [28]) from each participant median within the positive and negative pictures’ categories and from each picture median within each experimental group, and, finally, f) 20% trimmed mean from each participant average.

The findings pointed to a trend of results whereby positive pictures were constantly evaluated as more positive when touched from the top of the TM. From the other side, negative pictures tended to less negative evaluations when touched from the bottom of the TM, reaching this tendency the higher effect in analyses that fitted better residuals distribution (i.e., valence ratings’ exclusion of 2SDs; see S1 Table). In summary, the analyses performed following a multiverse approach support the hypothesis that the emotional pictures were evaluated as more positive when they were touched and moved from their congruent vertical locations.

## General discussion

The aim of the present study was to investigate whether interacting with valence-laden pictures in a touchscreen device would affect their perceived valence. Specifically, it was examined how moving these pictures upwards or downwards would influence their valence evaluations when they stick directly to the hand. To do so, participants touched the pictures and subsequently moved them with their dominant right hand either from the bottom to the top or from the top to the bottom of a touchscreen monitor. If touching the pictures while performing the moving gesture is irrelevant for judging the pictures’ valence, similar results than in the study by Sasaki, Yamada, and Miura [13] were expected.

Accordingly, the moving direction should influence the evaluation of the picture, regardless of the valence of the picture. That means that both, positive and negative pictures should be evaluated more positively when moved upwards and more negatively when moved downwards. However, if touching the pictures during performing the moving gesture is relevant for judging the pictures’ valence, it was expected that the valence of the picture that sticks to the hand get into the focus of attention together with the valence of its location (i.e., upper location–positive; lower location–negative), thus promoting a link between the valence of the picture and the valence associated with its location. This link can either represent a match or a mismatch between the picture valence and the valence of its location. Based on former findings it was assumed that a match between the valences of pictures and their location would be evaluated more positively compared to a mismatch [16]. Accordingly, it was hypothesized that positive and negative pictures would be evaluated more positively if they were moved from a matching location with regard to their valences. In other words, moving positive (negative) pictures on a touchscreen from top-to-bottom (bottom-to-top) would result in a relatively positive evaluation due to the congruent match between the valence of the picture and the valence of the location of the touch point. Conversely, moving positive (negative) pictures from bottom-to-top (top-to-bottom) would result in a relatively negative evaluation due to the incongruent match between the valence of the picture and the valence of the location of the touch point.

The pattern of results in the present study supports this hypothesis. Specifically, the findings support the notion that pictures were more positively evaluated when they were touched in a location whose associated valence category matches the valence category of the moved picture (i.e., positive picture–starting point of the moving gesture in the upper “positive” space and negative picture–starting point of the moving gesture in the lower “negative” space of a touchscreen). In contrast, valence evaluations of both positive and negative pictures are, rather, negative when the associated valence of the starting point of the moving gesture does not match the valence category of the moved picture (i.e., positive picture–starting point of the movement in the lower space and negative picture–starting point of the movement in the upper space of a touchscreen).

According to these findings, one may conclude that touching emotional pictures directly while performing vertical moving gestures results in different valence evaluations of the pictures than when moving the pictures vertically without touching them, as is the case in the earlier study by Sasaki et al., [13].

Specifically, the results of the study by Sasaki and colleagues indicated that both positive and negative pictures were evaluated more positively following upward movements and more negatively evaluated following downward movements, thus, suggesting that the movement direction influenced the valence evaluation of the pictures. The authors explained this effect with a space-valence mapping associating positive valence with an upward and negative valence with a downward direction in vertical space. According to this mapping, one can say that ending the movement in the positive upper space implies a *“positive influence”* on the valence evaluation of the picture whereas ending the movement in the negative lower space implies a “*negative influence*” on the valence evaluation of the pictures, independent of their valence category. However, the study of Sasaki et al. did not examine moving gestures where the picture sticks directly to the acting hand, which is standard when interacting with a multi-touch device.

But why should moving a picture sticking to the hand lead to a different influence on the valence evaluation than a picture, which is moved without touching directly? Earlier studies have shown that hand proximity influences attention deployment by showing that events that occur in the proximity of an individual’s hand are better attended than those taking place in distal places to the hand [29]. This suggests that hand proximity towards a stimulus influences the attention deployed to this stimulus and its location, increasing the depth of its processing, concretely the visuospatial processing (e.g., processing of pictures in its location; cf. [14]). It has been argued that this deeper visuospatial processing is due to a visual processing that seems to benefit from the inhibition of attentional disengagement and from an attentional prioritization of the space near the hands. Accordingly, information processing near the hand is improved [30] and leads to a prioritization of the established link between the valences of the picture and its location, which, compared to the valence of moving direction, then determines the bias in subsequent valence evaluations.

## Conclusions

Considering the widespread use of touchscreen devices the results of this study extends earlier results by an important point: Touching valence-laden pictures is relevant for their subsequent emotional processing. Furthermore, it can also be concluded that results obtained in traditional experimental environments using a computer screen and/or reacting to stimuli using a keyboard or an external key, cannot be automatically compared to the results obtained using newer experimental environments as, for example, touchscreen devices.

## Supporting information

S1 Table Multiverse analyses approach. LMM’s results summary.
(DOCX)
