# Supplementary material for: Valence-space associations in touchscreen interactions: Valence match between emotional pictures and their vertical touch location leads to pictures' positive evaluation
Source: PLoS One. 2018 Jul 18;13(7):e0199972. doi: 10.1371/journal.pone.0199972 (PMC6051585; doi:10.1371/journal.pone.0199972)
Supplement: S1 Table — LMM’s results summary. (DOCX) [file pone.0199972.s001.docx]

| LMM  (%trial exclusion) | AIC  (AIC random) | Main effects | | Interaction | |
| --- | --- | --- | --- | --- | --- |
|  |  | Movement (M) | Valence Category (VC) | M*VC | Contrasts [Cohen’s *d* effect size] |
| Initial LMM | 7718.15  (8790.61) | *F*(1,59) = .015, *p* = 0.903 | *F*(1,89) = 314, *p* < 0.001 | *F*(1,2281) = 15.7, *p* = 0.001 | *t*_(+)_ (108) = 2.36, *p* = 0.02 [*d* = .54] |
|  |  |  |  |  | *t*_(-)_ (129) = 2.02, *p* = 0.045 [*d* = .36] |
| LMM Box-Cox  transformation | 2936.11  (3984.2) | *F*(1,68) = .02, *p* = 0.887 | *F*(1,75) = 362, *p* < 0.001 | *F*(1,1979) = 12.2, *p* = 0.001 | *t*_(+)_ (81) = 2.05, *p* = 0.043 [*d* = .47] |
|  |  |  |  |  | *t*_(-)_ (196) = 1.83, *p* = 0.069 [*d* = .24] |
| LMM 3SD (1.17%) | 7255.64  (8352.9) | *F*(1,59) = .15, *p* = 0.697 | *F*(1,79) = 355, *p* < 0.001 | *F*(1,2263) = 16.3, *p* = 0.001 | *t*_(+)_ (126) = 2.5, *p* = 0.013 [*d* = .56] |
|  |  |  |  |  | *t*_(-)_ (110) = 1.92, *p* = 0.058 [*d* = .35] |
| LMM 2.5SD (2.87%) | 6880.77  (7913.1) | *F*(1,59) = .206, *p* = 0.652 | *F*(1,76) = 425, *p* < 0.001 | *F*(1,2221) = 17, *p* = 0.001 | *t*_(+)_ (129) = 2.59, *p* = 0.011 [*d* = .59] |
|  |  |  |  |  | *t*_(-)_ (109) = 1.92, *p* = 0.057 [*d* = .35] |
| LMM 2SD  (7%) | 6238.77  (7139.22) | *F*(1,59) = .018, *p* = 0.895 | *F*(1,77) = 563, *p* < 0.001 | *F*(1,2115) = 15.07, *p* = 0.001 | *t*_(+)_ (129) = 2.17, *p* = 0.032 [*d* = .41] |
|  |  |  |  |  | *t*_(-)_ (104) = 2.05, *p* = 0.043 [*d* = .44] |
| LMM 2.5 MAD (4.9%) | 6654.13  (7623.5) | *F*(1,58) = .86, *p* = 0.357 | *F*(1,83) = 482, *p* < 0.001 | *F*(1,2179) = 18.73, *p* = 0.001 | *t*_(+)_ (116) = 3.05, *p* = 0.03 [*d* = .86] |
|  |  |  |  |  | *t*_(-)_ (104) = 1.05, *p* = 0.136 |
| LMM (20% trimmed mean) | 5309.78  (6043.4) | *F*(1,58) = .142, *p* = 0.707 | *F*(1,82) = 597, *p* < 0.001 | *F*(1,1799) = 18.26, *p* = 0.001 | *t*_(+)_ (113) = 2.43, *p* = 0.017 [*d* = .55] |
|  |  |  |  |  | *t*_(-)_ (92) = 1.87, *p* = 0.065 [*d* = .36] |

Multiverse analysis approach. The table summarize the results of Linear Mixed Models (LMM) on the data after considering different adjustments on extreme observations. Contrasts of the interactions are shown for positive pictures *t*(+) and negative pictures *t*(-).
